# Detection of β-Lactamase Residues in Milk by Sandwich ELISA

**DOI:** 10.3390/ijerph10072688

**Published:** 2013-06-28

**Authors:** Wenbing Wang, Liqiang Liu, Liguang Xu, Wei Ma, Hua Kuang, Chuanlai Xu

**Affiliations:** State Key Laboratory of Food Science & Technology, School of Food Science & Technology, Jiangnan University, Wuxi 214122, China; E-Mails: wenbin66@yeah.net (W.W.); raxray@gmail.com (L.L.); xuliguang2006@yahoo.com.cn (L.X.); mawei209@126.com (W.M.); xcl@jiangnan.edu.cn (C.X.)

**Keywords:** β-lactamase, monoclonal antibody, sandwich ELISA, milk, detection

## Abstract

β-Lactamase residues in milk represent a public health risk. The cylinder plate detection method, which is based on bacterial growth, is laborious and time consuming. In this study, 15 monoclonal antibodies (mAbs) were selected against Temoneira (TEM) 1 β-lactamase. A sandwich enzyme-linked immunosorbent assay (ELISA) based on an optimum mAb pair was developed and validated for the detection of β-lactamase. The limit of detection and linear dynamic range of the method were 4.17 ng/mL and 5.5–100 ng/mL, respectively. β-Lactamase recovery in pure milk was 96.82–103.13%. The intra- and inter-assay coefficients of variation were 6.21–7.38% and 12.96–13.74%, respectively. Our developed sandwich ELISA can be used as a rapid detection method of β-lactamase in milk.

## 1. Introduction

The presence of antibiotic residues in milk is a long-standing problem in the dairy industry. Antibiotic residues emerge from antibiotics given to lactating cattle infected with bacterial mastitis or added to raw milk to prevent bacterial growth. Due to serious public health risks [1,2,3,4,5], the use of antibiotics in milk has been strictly controlled. For example, in the European Union, the maximum residue limit (MRL) of antibiotics in milk powder such as benzylpenicillin, ampicillin, and amoxicillin is 4 μg/kg.

Bacterial resistant to β-lactam antibiotics (e.g., penicillin and cephalosporin) produce β-lactamases (EC 3.5.2.6, BLA), which degrade these antibiotics. According to their molecular structure, β-lactamases are divided into four classes: class A (serine penicillinases), class B (metalloenzymes), class C (serine cephalosporinases), and class D (oxacillin-hydrolyzing serine β-lactamases) [6]. By degrading β-lactam antibiotics, β-lactamases confer drug resistance to bacteria. In medicine, β-lactamases are commonly used to remove antibiotic residues from medical instruments and thus prevent allergic reactions in patients. Korycka-Dahl *et al.*, who used β-lactamase to degrade penicillin in milk, reported that the safety of β-lactamase-treated dairy products for human consumption remains to be established [3]. Because of the limited data on the safety of β-lactamase and on the degradation products of antibiotics in milk, β-lactamase has not been used as a food additive.

In the past years, penicillinase, which is a specific type of β-lactamase, was illegally used in China to degrade residual β-lactam antibiotics in milk. Although the presence of endogenous β-lactamase in milk is possible, positive-test results of β-lactamase in packaged milk and negative-test results of β-lactamase in raw milk are indicative of illegal β-lactamase use.

Currently, there is no standard method to efficiently detect β-lactamase in dairy products. The conventional cylinder plate method [7], which is based on penicillin G-sensitive bacteria, is sensitive (sensitivity of 4 U/mL) and relatively easy. However, this method is time consuming (18 h) and therefore not suitable as a rapid detection test. Other methods such as the acidometric and iodometric methods, chromogenic cephalosporin spot and phenotypic tests, Etest, and PCR are traditionally used to detect β-lactamases in bacterial strains [8,9,10,11,12,13,14,15]. The iodometric method is affected by the matrix and has low sensitivity for detecting β-lactamase in milk samples.

Alternative methods such as the rapid resolution liquid chromatography-tandem mass spectrometry (RRLC-MS/MS) and matrix-assisted laser desorption/ionization Fourier transform mass spectrometry (MALDI-FTMS) have been used to detect β-lactamase in milk [16,17]. These methods yield consistent results with the cylinder plate method. Additionally, the simple centrifugation step involved during sample preparation makes these methods less laborious compared to others. However, these methods are quite expensive.

Immunologic methods such as sandwich ELISA are effective for the detection of proteins and bacteria [18,19,20,21,22]. Suitable antibodies for the detection of β-lactamases by ELISA have been developed. Hujer *et al.* developed polyclonal rabbit antibodies against SHV-1 and CMY-2-lactamases [23,24,25]. According to the authors, the developed sandwich ELISA for each lactamase was specific, *i.e.*, it did not react with other types of β-lactamases. Morin *et al.* developed and characterized 28 murine monoclonal antibodies (mAbs) against plasmid-mediated TEM-1 β-lactamase. Of the 28 mAbs, 16 monoclonal antibodies had cross-reactivity with TEM-2, TLE-1, and, to some degree, SHV-1 [22]. Even though mAbs have been produced, no analytical method has been developed. In this study, we produced mAbs against TEM-1 β-lactamase (*i.e.*, parental penicillinase) and developed a sandwich ELISA for the detection of this penicillin degrader.

## 2. Experimental Section

### 2.1. Chemicals and Materials

TEM-1 β-lactamase was purchased from Aladdin Industrial Inc. (Shanghai, China). Complete and incomplete Freund’s adjuvant and enzyme immunoassay-grade horseradish peroxidase-labeled goat anti-mouse immunoglobulin were obtained from Sigma (St. Louis, MO, USA). Both 3,3′,5,5′-tetramethylbenzidine (TMB) and horseradish peroxidase (HRP) were purchased from Aladdin Chemistry Co., Ltd. (Shanghai, China). Gelatin was obtained from Beijing Biodee Biotechnology Co., Ltd. (Beijing, China). Pure milk was purchased at a local supermarket. Other reagents and chemicals were obtained from the National Pharmaceutical Group Chemical Reagent Co., Ltd. (Shanghai, China).

### 2.2. Solutions

The solutions used in the study included a coating buffer (0.01 M sodium carbonate buffer, pH 9.6), blocking buffer (0.2% w/v gelatin in coating buffer), 0.01 M phosphate-buffered saline (PBS, pH 7.4), washing buffer (PBS containing 0.05% v/v Tween 20), antibody dilution buffer (PBS containing 0.1% w/v gelatin and 0.05% v/v Tween 20), stop buffer (2 M sulfuric acid), and substrate solution. The substrate solution was prepared by mixing 2 mL of 0.06% (w/v) TMB in glycol with 10 mL of 0.1 M citrate phosphate buffer (pH 5.0) containing 1.8 μL of 30% hydrogen peroxide.

### 2.3. Antibodies and Conjugated Antibodies

Female BALB/c mice (6–8 weeks old) were prepared for immunization. First, the mice were immunized by a normal subcutaneous procedure using a series of three doses [18]. The doses were 100, 100, and 50 μg β-lactamase. Seven days after the third immunization, the immune responses of the mice were measured by indirect ELISA. The mouse with the highest titer was sacrificed and its spleen was fused with Sp2/0 murine myeloma cells. The target cells were selected by indirect ELISA and obtained by limiting dilution. The mAbs were purified by the caprylic acid-ammonium sulfate precipitation method and then conjugated to HRP as described [20]. Antibodies that conjugated to HRP were characterized by direct ELISA.

### 2.4. Sandwich ELISA

Ninety-six-well microplates were coated with anti-β-lactamase mAb diluted in coating buffer (100 μL/well) and subsequently incubated at 4 °C overnight. Following incubation, the wells were washed three times with washing buffer; the free binding sites in the wells were blocked with blocking buffer (220 μL/well) at 37 °C for 2 h. Following another washing step, 100 μL of a serially diluted β-lactamase standard solution or sample extract solution was added to each well, and the microplate was incubated at 37 °C for 1 h. Subsequently, 100 μL of HRP-labeled anti-β-lactamase mAb was added to each well, and the plate was incubated for 1 h at 37 °C. After washing the plate five times, 100 μL of TMB substrate solution was added to each well and allowed to react with the labeled mAb at 37 °C for 15 min in the dark. The reaction was stopped by adding 2 M sulfuric acid (50 μL/well). Absorbance was measured at 450 nm in a microplate reader. All measurements were performed in triplicate.

### 2.5. Indirect ELISA

Indirect ELISA was carried out to detect the serum titers and to screen the hybridoma cell lines. ELISA plates containing 100 μL/well of β-lactamase in coating buffer were incubated at 37 °C for 2 h. Following incubation, the plates were washed three times with washing buffer, blocked with blocking buffer (220 μL/well), and incubated for 2 h at 37 °C. After washing the plates, cell supernatant or mouse serum diluted with antibody dilution buffer was added to the wells (100 μL/well). The microplates were incubated at 37 °C for 30 min. After washing the plates three times, HRP-labeled goat anti-mouse immunoglobulin, which was diluted with antibody dilution buffer at a ratio of 1:3,000, was added (100 μL/well), and the plates were incubated at 37 °C for 30 min. After washing the plates four times, 100 μL of freshly prepared TMB substrate solution was added to each well and allowed to react at 37 °C for 15 min in the dark. The reaction was stopped with the addition of 2 M sulfuric acid (50 μL/well). Absorbance was measured at 450 nm in a microplate reader.

### 2.6. Pairwise Interaction Analysis

To select the best mAb pair for the sandwich ELISA of β-lactamase, mAbs from 15 cell lines were labeled with HRP and analyzed by sandwich ELISA. One anti-β-lactamase mAb was utilized as the capture antibody to pair with the other 14 anti-β-lactamase mAbs, which were labeled with HRP and used as the detection antibodies. Based on our experience, 4 μg/mL of capture antibody and 2 μg/mL of detection antibody yield optimum results. These concentrations were therefore used in our experiments. The positive control was added with 100 ng/mL β-lactamase in PBS and the negative control was added with 0.01 M PBS.

### 2.7. Selection of Optimum mAb Pairs for β-Lactamase Detection

To select the optimum pair, coupled pairs obtained in pairwise interaction analyse were further tested in sandwich ELISA. PBS and serially diluted β-lactamase in PBS were added to a microplate as negative control and standard, respectively. Standard curves of different pairs were generated and compared.

### 2.8. Assessment of Cross-Reactivity of the Developed Sandwich ELSA

Bush type IV β-lactamase, alkaline phosphatase, β-D-glucuronidase, casein, OVA, and BSA were tested by the developed sandwich ELISA. All reactants were diluted in PBS containing 0.01% Tween 20.

### 2.9. Sample Preparation

Pure milk, purchased at a local market, was β-lactamase free based on the cylinder plate method. Different volumes of β-lactamase (1 mg/mL) were added to milk resulting in different β-lactamase concentrations. The milk samples were centrifuged at 3,500 × g at 15 °C for 10 min. After centrifugation, milk fat was removed from the supernatant, and the milk was immediately analyzed. 

### 2.10. Analysis of β-Lactamase-Spiked Milk by Sandwich ELISA

Milk was spiked with either 10 ng/mL or 40 ng/mL of purified β-lactamase. The spiked milk samples were subjected to the same treatment described in Section 2.9. The β-lactamase concentration in the spiked milk sample was calculated based on a standard curve.

## 3. Results and Discussion

### 3.1. Production of Anti-β-Lactamase mAbs

Seven days after cell fusion, the anti-β-lactamase mAbs in the supernatant from the cell culture plates were detected by indirect ELISA. Target cells for β-lactamase were selected and sub-cloned three times by limiting dilution. After selection, 15 hybridoma cell lines, labeled mAb1 through mAb15, were obtained. These cells were intraperitoneally injected into mice, and the ascitic fluid produced was removed for preparation of the mAbs.

### 3.2. Pairwise Interaction Analysis of mAbs

The ratios between the OD_450_ of the positive control and the OD_450_ of the negative control are shown in Table 1.

**Table 1:** Sandwich ELISA for pair-wise interaction analysis (P/N value).

D mAb	Capture mAb														
	1	2	3	4	5	6	7	8	9	10	11	12	13	14	15
1-HRP		12.29	2.07	3.48	7.30	7.77	5.72	7.25	2.00	9.85	8.29	2.59	3.56	1.84	1.18
2-HRP	11.88		2.36	15.28	6.41	5.24	6.05	5.96	4.47	3.09	3.74	3.72	4.78	3.26	1.43
3-HRP	3.57	1.99		4.21	1.19	1.38	1.11	1.41	1.20	2.16	1.92	1.07	1.34	1.27	0.92
4-HRP	5.95	14.70	4.51		12.56	12.81	10.60	10.73	2.78	13.09	9.11	3.84	4.69	1.93	0.91
5-HRP	9.70	4.77	1.38	11.35		3.01	1.96	2.75	1.86	5.13	2.52	1.37	2.29	2.10	1.19
6-HRP	5.18	1.60	1.34	5.73	2.16		2.11	1.78	1.60	1.17	1.42	1.37	1.72	1.67	1.10
7-HRP	3.01	2.26	1.00	6.20	1.29	1.59		1.76	1.45	2.75	2.09	1.06	1.25	1.33	0.89
8-HRP	3.97	1.69	1.38	7.37	2.66	2.39	2.98		0.99	1.71	2.17	1.24	2.35	1.95	1.18
9-HRP	0.96	1.82	0.89	0.90	1.27	1.33	1.19	1.14		2.14	1.10	0.77	1.01	1.01	1.01
10-HRP	11.41	5.16	2.41	13.10	4.93	5.23	5.80	4.95	3.65		3.97	3.80	5.06	3.71	1.79
11-HRP	10.56	5.09	2.53	15.55	6.10	4.81	6.35	5.06	3.81	2.76		4.47	4.22	3.25	1.58
12-HRP	2.34	2.73	1.06	2.97	1.99	2.24	1.64	1.48	1.07	3.45	2.03		1.54	1.27	0.91
13-HRP	3.13	4.64	1.21	4.66	2.25	2.57	2.00	2.78	1.47	3.93	2.88	1.77		1.49	1.31
14-HRP	3.86	9.41	2.19	3.63	4.79	6.10	5.33	5.11	1.74	10.96	7.80	2.75	1.13		0.92
15-HRP	1.63	2.83	1.19	1.19	1.93	1.77	1.59	1.50	1.08	4.32	2.11	1.01	3.95	1.67	

These results indicate that many candidate mAb pairs were obtained; mAbs 1, 2, 4, and 10 contributed to most of the pairs. When used as a detection antibody, mAb 4 can be paired with mAbs 1–13. This result suggests that mAb 4 recognizes a unique epitope, which is far from the epitopes of the other mAbs. Conversely, mAbs 6, 7, and 8 did not couple well with other mAbs when used as detection antibodies. Because the antibodies that conjugated with HRP were characterized by direct ELISA, this result may be attributed to steric hindrance between the capture and detection antibodies. This difference suggests that relatively more cell lines are essential for the pairwise interaction analysis.

### 3.3. Selection of an Optimum mAb Pair for β-Lactamase Detection

**Figure 1:**
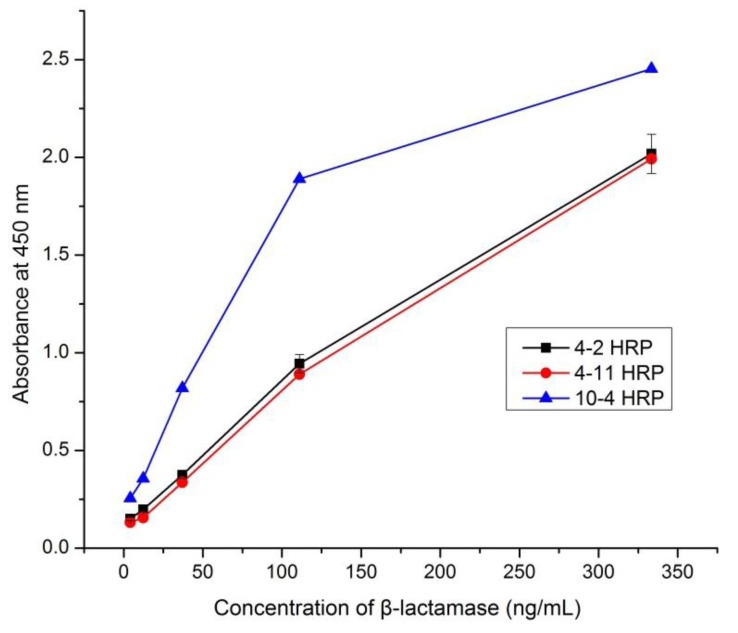
Comparison of running curves between different pairs.

Figure 1 shows the running curves of three representative pairs in PBS: 4-2 HRP, 4-11 HRP, and 10-4 HRP. 4-2 HRP and 4-11 HRP were the pairs with the highest ratio and absorbance values at 450 m (absorbance values of other mAb pairs is not shown). The results indicate that the sandwich ELISA with 10-4 HRP is more sensitive than that with 4-2 HRP or 4-11 HRP. This result is consistent with our previous findings that pairs with the highest ratio or absorbance may not guarantee the optimum pair [20]. Therefore, mAb 10 was used as the capture antibody and mAb 4 was used as the detection antibody; mAbs 10 and 4 were designated as the optimum mAb pair.

### 3.4. Development of mAb-Based Sandwich ELISA for the Detection of β-Lactamase

**Figure 2:**
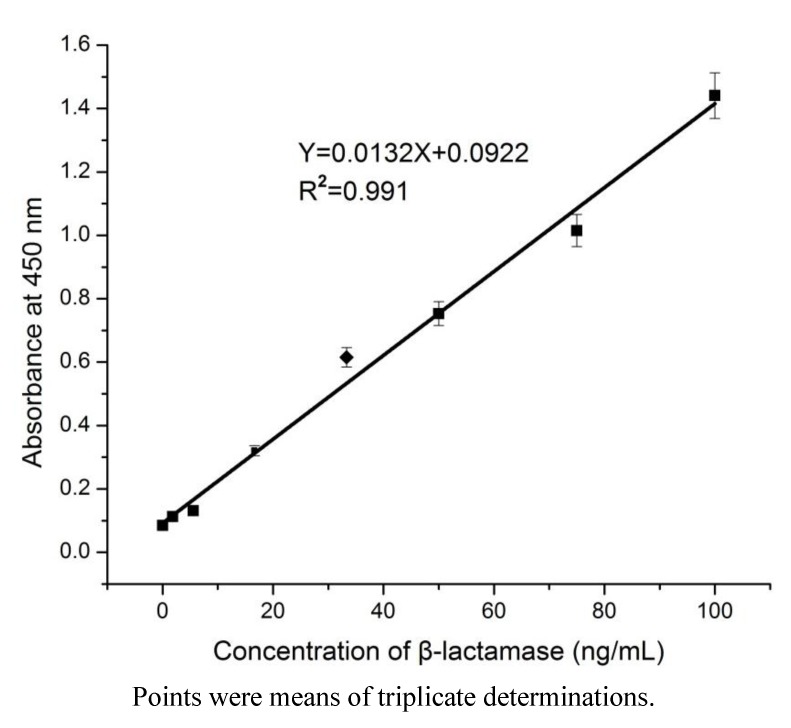
Calibration curve (in pure milk) for the detection of β-lactamase.

**Figure 3:**
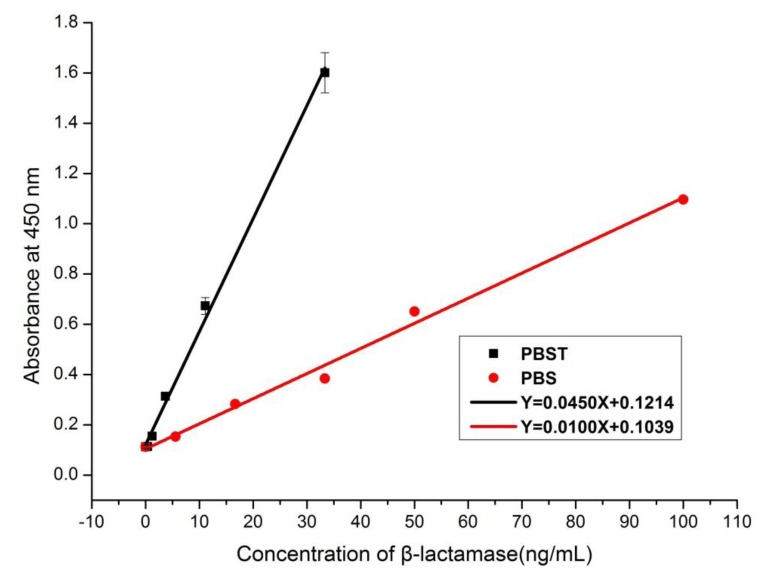
Running curves in PBS and PBS containing 0.1% Tween.

Based on the optimum mAb pair, an mAb-based sandwich ELISA for the detection of β-lactamase was developed. The optimum concentration of the capture antibody and the dilution factor of the detection antibody were 5 μg/mL and 2,000, respectively. Commercial β-lactamase was used as a standard and was added to pure milk, PBS, or PBS containing 0.01% Tween 20. Figure 2 shows the calibration curve of different concentrations of β-lactamase in pure milk. LOD (limit of detection) was calculated by multiplying SD (standard deviation) of the blank absorbance value by 3 and dividing the result by the slope of the standard curve. Figure 3 shows the running curves of different concentrations of β-lactamase in PBS or PBS containing 0.01% Tween 20 (PBST). The LOD of β-lactamase in PBST was 0.56 ng/mL, which was lower (*i.e.*, more sensitive) than that of β-lactamase in PBS or in pure milk. The sensitivity of this method was enhanced by the presence of Tween 20 in the dilution buffer. However, a substantial difference of the OD in PBST and the OD in pure milk was obtained when β-lactamase in PBST was used as the calibration curve. In this case, the recovery of 100 ng/mL β-lactamase in pure milk was only 59% when PBST was used as the dilution buffer. The curve obtained from β-lactamase in PBS was not stable; however, it was more comparable to that obtained from β-lactamase in milk. Therefore, β-lactamase in pure milk was used as the calibration curve for the detection of β-lactamase. The LOD and the linear dynamic range of this method were 4.17 ng/mL and 5.5–100 ng/mL, respectively.

### 3.5. Method Specificity

**Figure 4:**
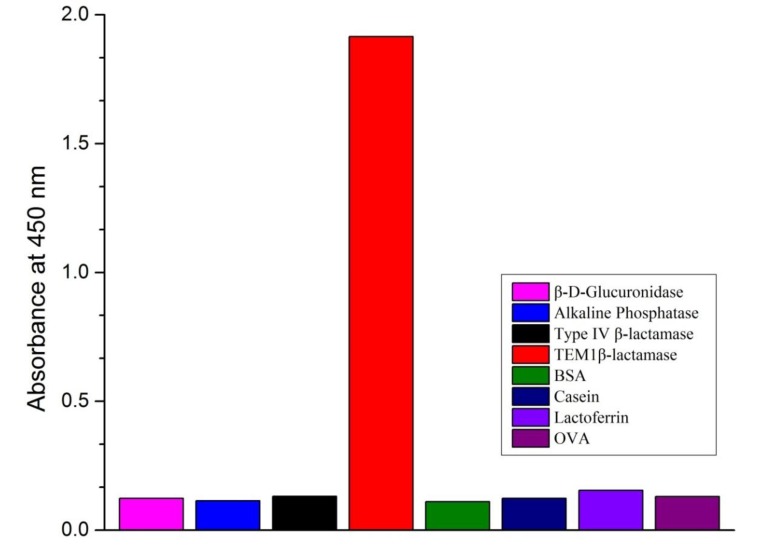
Cross-reactivities of the ELISA toward some enzymes from bacteria and some common proteins. Concentration of TEM1 β-lactamase is 50 ng/mL. Concentration of other reactants is 10 ug/mL.

As mentioned previously, β-lactamases are divided into four categories (class A, B, C, and D) based on their amino acid sequence. Several researchers have reported that different β-lactamases may share a certain degree of homology [22,23,24]. Because several types of proteins are present in milk, it is crucial to assess any cross-reactivity in milk using the developed β-lactamase detection method. Figure 4 shows that the OD of TEM1 β-lactamase at 50 ng/mL was ~2. On the other hand, the ODs of other cross-reactants at 10 μg/mL were <0.16. Lactoferrin, which had the highest OD among the cross-reactants, had <0.01% cross-reactivity in the developed sandwich ELISA. The molecular structure of type IV β-lactamase is not known. The low cross-reactivity of type IV β-lactamase suggests that our developed sandwich ELISA was very specific for TEM1 β-lactamase. This finding was possible because sandwich ELISA is based on two monoclonal antibodies, each of which specifically recognizes one epitope. Other studies have reported similar results. In a previous study, we developed a highly sensitive sandwich ELISA for the detection of staphylococcal enterotoxin A (SEA). The mAb-based sandwich ELISA was very specific for SEA; however, staphylococcal enterotoxins do share a certain degree of homology. The low cross-reactivity with similar proteins, especially milk proteins, confirms the specificity of this method. Furthermore, the results may explain the low background and insignificant matrix effects obtained in pure milk.

### 3.6. Recovery and Validation Tests

The intra- and inter-assay recoveries and coefficient of variation (CV) of the developed sandwich ELISA were assessed. The intra-assay recovery was determined by analyzing each β-lactamase concentration six times per run at one time, whereas the inter-assay recovery was determined by analyzing each concentration at six different times. Table 2 shows that the intra- and inter-assay recoveries for 10 ng/mL and 40 ng/mL β-lactamase were 103.13–100.22% and 96.82–97.23%, respectively. The intra- and inter-assay CVs for 10 ng/mL and 40 ng/mL β-lactamase were 6.21–7.38% and 13.74–12.96%, respectively. These results indicate that our developed sandwich ELISA is accurate and reliable for detecting TEM 1 β-lactamase in milk.

**Table 2:** Recovery of sandwich ELISA for β-lactamase.

Sparked level (ng/mL)	Intra-assay (n = 6)			Inter-assay (n = 6)		
	Mean ± SD	Recoveries	CV (%)	Mean ±SD	Recoveries	CV (%)
	(ng/mL)	(%)		(ng/mL)	(%)	
40	40.086 ± 2.963	100.21	7.38	38.886 ± 5.035	97.22	12.96
10	10.313 ± 0.6422	103.13	6.21	9.682 ± 1.326	96.82	13.74

## 4. Conclusions

β-Lactamase residues in milk represent a public health risk. Furthermore, it is a challenge to measure and detect illegal food additives. In this study, we produced 15 high affinity monoclonal antibodies of TEM 1 β-lactamase by routine immunization, fusion, and selection. Following the selection of an optimum mAb pair by the pairwise interaction assay, a sandwich ELISA for β-lactamase detection was developed. The LOD and linear dynamic range of the method were 4.17 ng/mL and 5.5–100 ng/mL, respectively. Converted to units of enzyme activity, the sensitivity of our developed sandwich ELISA was 4.17 × 10^−3^ U/mL (1,000 units/mg enzyme). On the other hand, the sensitivity of the conventional cylinder plate method is 4 U/mL. The intra- and inter-assay results revealed that this method was both accurate and reliable. Our developed sandwich ELISA can be used as a rapid detection method of β-lactamase residues in large volumes of milk. Further studies should focus on other molecular types of β-lactamase to characterize the specificity of this method.

## References

[1] Goldman E. (2004). Antibiotic abuse in animal agriculture: Exacerbating drug resistance in human pathogens. Hum. Ecol. Risk Assess..

[2] Khan S.A., Feroz F., Noor R. (2013). Study of extended-spectrum β-lactamase-producing bacteria from urinary tract infections in Bangladesh. Tzu Chi Med. J..

[3] Korycka-Dahl M., Richardson T., Bradley R.L. (1985). Use of microbial β-lactamase to destroy penicillin added to milk. J. Dairy Sci..

[4] Luvsansharav U.O., Hirai I., Nakata A., Imura K., Yamauchi K., Niki M., Komalamisra C., Kusolsuk T., Yamamoto Y. (2012). Prevalence of and risk factors associated with faecal carriage of CTX-M beta-lactamase-producing Enterobacteriaceae in rural Thai communities. J. Antimicrob. Chemother..

[5] Shea K.M. (2003). Antibiotic resistance: What is the impact of agricultural uses of antibiotics on children’s health?. Pediatrics.

[6] Bush K. (1989). Characterization of beta-lactamases. Antimicrob. Agents Chemother..

[7] Cui S.H., Li J.Y., Hu C.Q., Jin S.H., Ma Y. (2007). Development of a method for the detection of beta-lactamases in milk samples. J. Aoac Int..

[8] Doi Y., Paterson D.L. (2007). Detection of plasmid-mediated class C beta-lactamases. Int. J. Infect. Dis..

[9] O’Callaghan C.H., Morris A., Kirby S.M., Shingler A.H. (1972). Novel method for detection of β-Lactamases by using a chromogenic cephalosporin substrate. Antimicrob. Agents Chemother..

[10] Perez-Perez F.J., Hanson N.D. (2002). Detection of plasmid-mediated AmpC β-lactamase genes in clinical isolates by using multiplex PCR. J. Clin. Microbiol..

[11] Robberts F.J., Kohner P.C., Patel R. (2009). Unreliable extended-spectrum β-lactamase detection in the presence of plasmid-mediated AmpC in *Escherichia coli* clinical isolates. J. Clin. Microbiol..

[12] Ruppe E., Bidet P., Verdet C., Arlet G., Bingen E. (2006). First detection of the Ambler class C 1 AmpC beta-lactamase in Citrobacter freundii by a new, simple double-disk synergy test. J. Clin. Microbiol..

[13] Sanguinetti M., Posteraro B., Spanu T., Ciccaglione D., Romano L., Fiori B., Nicoletti G., Zanetti S., Fadda G. (2003). Characterization of clinical isolates of *Enterobacteriaceae* from Italy by the BD phoenix extended-spectrum β-lactamase detection method. J. Clin. Microbiol..

[14] Selepak S.T., Witebsky F.G. (1984). beta-Lactamase detection in nine staphylococcal species. J. Clin. Microbiol..

[15] Walsh T.R., Bolmstrom A., Qwarnstrom A., Gales A. (2002). Evaluation of a new etest for detecting metallo-β-lactamases in routine clinical testing. J. Clin. Microbiol..

[16] Sun H.W., Li H., Zhang J.X., Zhou Z. (2010). Qualitative analysis of active beta-lactamases in milk samples by rapid resolution liquid chromatography-tandem mass spectrometry. Chinese J. Anal. Chem..

[17] Xu Z., Wang H.Y., Huang S.X., Wei Y.L., Yao S.J., Guo Y.L. (2010). Determination of beta-lactamase residues in milk using matrix-assisted laser desorption/ionization fourier transform mass spectrometry. Anal. Chem..

[18] Deng X.F., Liu L.Q., Ma W.W., Xu C.L., Wang L.B., Kuang H. (2012). Development and validation of a sandwich ELISA for quantification of peanut agglutinin (PNA) in foods. Food Agr. Immunol..

[19] Feng M., Yong Q., Wang W., Kuang H., Wang L., Xu C. (2012). Development of a monoclonal antibody-based ELISA to detect *Escherichia coli* O157:H7. Food Agr. Immunol..

[20] Kuang H., Wang W., Xu L., Ma W., Liu L., Wang L., Xu C. (2013). Monoclonal antibody-based sandwich ELISA for the detection of staphylococcal enterotoxin A. Int. J. Environ. Res. Public Health.

[21] Peng J., Meng X., Deng X., Zhu J., Kuang H., Xu C. (2013). Development of a monoclonal antibody-based sandwich ELISA for the detection of ovalbumin in foods. Food Agr. Immunol.

[22] Morin C.J., Patel P.C., Levesque R.C., Letarte R. (1987). Monoclonal antibodies to TEM-1 plasmid-mediated beta-lactamase. Antimicrob. Agent. Chemother..

[23] Hujer A.M., Page M.G.P., Helfand M.S., Yeiser B., Bonomo R.A. (2002). Development of a sensitive and specific enzyme-linked immunosorbent assay for detecting and quantifying CMY-2 and SHV β-lactamases. J. Clin. Microbiol..

[24] Bauernfeind A., Stemplinger I., Jungwirth R., Ernst S., Casellas J.M. (1996). Sequences of beta-lactamase genes encoding CTX-M-1 (MEN-1) and CTX-M-2 and relationship of their amino acid sequences with those of other beta-lactamases. Antimicrob. Agent. Chemother..

[25] Hujer A.M., Keslar K.S., Dietenberger N.J., Bethel C.R., Endimiani A., Bonomo R.A. (2009). Detection of SHV beta-lactamases in Gram-negative bacilli using fluorescein-labeled antibodies. BMC Microbiol.

